# South African men and women living with HIV have similar distributions of pain sites

**DOI:** 10.4102/phcfm.v14i1.3114

**Published:** 2022-01-11

**Authors:** Antonia L. Wadley, Romy Parker, Vanessa A. Mukhuba, Andani Ratshinanga, Zipho Zwane, Peter R. Kamerman

**Affiliations:** 1 Brain Function Research Group, School of Physiology, Faculty of Health Sciences, University of the Witwatersrand, Johannesburg, South Africa; 2 Pain Management Unit, Department of Anaesthesia and Perioperative Medicine, Faculty of Health Sciences, University of Cape Town, Cape Town, South Africa

**Keywords:** pain, HIV, sex differences, pain sites, pain location, South Africa

## Abstract

**Background:**

No studies have investigated sex differences in the location and number of pain sites in people living with human immunodeficiency virus (HIV) (PLWH), despite evidence that women, in general, bear a greater burden of pain than men.

**Aim:**

To determine sex differences in the location and number of pain sites, and whether there were demographic or disease-related differences in the number of pain sites.

**Setting:**

South African tertiary hospital HIV clinics and a community healthcare centre

**Methods:**

We conducted a retrospective analysis of records from South African PLWH who had pain.

**Results:**

Of the 596 participant records, 19% were male (115/596) and the median number of pain sites for both sexes was 2 (interquartile range [IQR]: 1 to 3). Pain was most frequently experienced in the head (men: 12%, women: 38%), feet and ankles (men: 42%, women: 28%), abdomen (men = 19%, women = 28%) and chest (men = 20%, women = 20%). After correcting for multiple comparisons, males were less likely to experience headache than females (Fisher’s exact text, odds ratio [OR] = 0.23, 95% confidence interval [CI]: 0.12 – 0.42, *p* = 0.000). Pain at other body sites was experienced similarly between the sexes. There was no meaningful variation in the number of pain sites between the sexes (logistic regression, *p* = 0.157).

**Conclusion:**

A similar location and number of pain sites were experienced by male and female South African PLWH. The locations of pain sites were different from previous reports, however, suggesting that research into pain in PLWH cannot necessarily be generalised across cultures.

## Introduction

Globally, the burden of both acute and chronic pain is greater in women than in men.^1,2,3,4^ Women are more likely to suffer from a pain condition than men, they have lower tolerance to some modalities of experimental pain including models of cold, heat and pressure, and when both sexes experience clinical or experimental pain, women are likely to experience that pain at a greater intensity.^5,6^ Moreover, a large population-based study in Sweden reported that women were more likely to have pain across 10 pre-defined anatomical sites,^7^ whilst a Spanish population-based study reported that women were more likely to have headache than men, whilst men had greater prevalence of lower extremity pain.^8^ A recent national household survey found that South Africa too has a greater proportion of women living with chronic pain than men,^1^ and these women were more affected by abdominal and neck/shoulder pain than men.

Chronic pain is reported by over half of people living with HIV (PLWH) in their lifetimes.^9^ As there are 37 million PLWH globally,^10^ pain may affect more than 18 million of them. Pain in PLWH may be caused by immune dysregulation, HIV-related co-infections or be comorbid to the HIV.^11,12,13^ Pain in PLWH may be more likely to become chronic as a result of psychosocial factors such as exposure to HIV stigma and reduced social support, and there is also overlap between risk factors for contracting HIV and developing chronic pain.^13,14^ Pain in PLWH is commonly moderate to severe in intensity, often includes more than one pain site,^9^ and reduces quality of life and subjective daily functioning.^15,16^ However, there are few data on the sex differences in pain in PLWH, and where there are data, they are equivocal. For example, two studies have reported the prevalence of pain to be greater in women than men,^17,18^ one study reported pain to be more prevalent in men,^19^ whilst two other studies have reported no difference.^20,21^

Whilst there are these few studies on sex differences in the prevalence of pain in PLWH, there are no data on whether body sites affected by pain in PLWH differ between the sexes, both in terms of the number of pain sites and locations of these pains. As we try to understand the aetiologies of pain in PLWH, having a clearer picture on sex differences will help inform the direction of future work including whether there is a need to investigate hormonal, genetic, or sensing and modulation mechanisms.^22^

No studies have investigated sex differences in the location or number of pain sites in PLWH and pain. Thus, the aim of our study was to investigate sex differences in the pain sites of PLWH and pain. To achieve this aim, we retrospectively reviewed 596 records (481 women and 115 men) from five previous South African studies of PLWH who reported pain of any kind for at least the last week.^23,24,25,26,27^ Our objectives were to investigate whether there were sex differences in the location and number of pain sites and secondly, whether there were other demographic and disease-related differences in the number of pain sites. We hypothesised that there would be sex-dependent differences in the location of the pain sites, and women with pain and HIV would have a greater number of pain sites than men with pain and HIV.

## Methods

This study is a retrospective analysis of data from five previous studies from our collaboration, all conducted in South Africa. The studies have been published elsewhere,^23,24,25,26,27^ but none looked at sex-dependent association with the number and location of pain sites in those individuals with pain.

Inclusion criteria included being ≥ 18 years of age, having an HIV diagnosis and suffering from pain of any kind. Participants were not excluded for having previous trauma, chronic pain with onset prior to HIV diagnosis, comorbidities (such as diabetes mellitus or tuberculosis [TB]), or the concurrent use of analgesics. One study (see below: Validation of isiXhosa Brief Pain Inventory study) had the following additional inclusion criteria: female sex and being isiXhosa-speaking. That study also excluded those with moderate-to-severe intellectual disability or cognitive impairment and being > 40 years of age. An additional study (see section: Pain intervention study) excluded those with moderate-to-severe intellectual disability or cognitive impairment and as the intervention study involved an exercise component, also excluded non-ambulatory participants who did not fulfil American College of Sports Medicine (ACSM) Health/Fitness Facility Pre-participation Screening Questionnaire criteria.^28^

Presence of pain was determined using (1) the Brief Pain Inventory (BPI)^29^:

Throughout our lives, most of us have had pain from time to time, such as minor headaches, sprains, and toothaches. Have you had pain other than everyday types of pain in the past week?

And (2) the Wisconsin Brief Pain Questionnaire (WBPQ)^30^:

Throughout our lives, most of us have had pain from time to time, such as minor headaches, sprains, and toothaches. Have you had pain other than everyday types of pain in the past month?

If pain had been present in the last week/month, participants were asked where on their bodies they experienced pain (assessed using a body chart).

Depressive symptoms were extracted from studies in which they had been assessed using the Beck Depression Inventory (BDI) II.^31^ The scale has 21 questions, which are answered on a 4-point Likert scale 0, 1, 2 and 3. The maximum score is thus 63, with a higher score indicating greater depressive symptoms.

Demographic information including age, education, and employment status were recorded from participants during interviews. Participants’ medical information including any other medical conditions, cluster of differentiation 4 (CD4) T-cell count, and details of any antiretroviral therapy (ART) were recorded from participants’ medical files. Details of the studies included here follows.

### Pain intervention study (study site 1)

This study made use of the BPI and was one of three study sites contributing data to a multi-centre trial assessing the efficacy of a 6-week, peer-led exercise and education intervention for PLWH with chronic pain. Between October 2014 and 2015, 60 participants were recruited from the HIV clinic at Charlotte Maxeke Johannesburg Academic Hospital, Johannesburg, South Africa. All participants had had pain in the last week. Participants’ data at the time of recruitment were included in these analyses.

### Pain in urban versus rural people living with HIV study (study site 2)

The study made use of the WBPQ, and was a cross-sectional study that compared pain in the last month between PLWH living in an urban and a rural area.^23^ Data collection was conducted between March 2005 and July 2006. A total of 396 urban participants were recruited from the HIV Clinic at the Helen Joseph Hospital, Johannesburg, Gauteng, South Africa. In addition. 125 rural participants were recruited from Tintswalo Hospital, Bushbuckridge, Limpopo province, South Africa (rural site). In total, 239 of the 521 participants had had pain in the last month and were included in this study.

### Resilience study (study site 3)

The study made use of the BPI, and was a cross-sectional study investigating the relationship between physical activity, and chronic pain, and resilience in PLWH.^26^ Between September 2014 and March 2015, a convenience sample of 197 participants were recruited from the HIV clinic at Charlotte Maxeke Johannesburg Academic Hospital, Johannesburg, Gauteng, South Africa. Of the 197 participants, 99 had had pain in the last week, and were included in the current analyses.

### Validation of isiXhosa Brief Pain Inventory study (study site 4)

This study was a translation and validation of the BPI into isiXhosa. A convenience sample of 229 females living with HIV were recruited from a Community Health Centre (CHC) in Khayelitsha, Cape Town, South Africa between February 2010 and December 2010 and were asked about having pain in the last week. Of the total participants recruited at CHC, 148 had had pain in the last week and were included in the current analyses.

### Stigma study (study site 5)

This study made use of the BPI. This was a proof-of-concept study aimed at determining if there was an association between perceived HIV stigma, pain intensity and depressive symptoms in PLWH. Data collection ran between April 2015 and June 2015. A convenience sample of 50 participants were recruited from the HIV clinic at Charlotte Maxeke Johannesburg Academic Hospital, Johannesburg, South Africa. All participants had had pain in the last week.

### Pain sites coding process

All body charts from the studies were re-coded by two independent coders. Using a standardised template, the coders recorded the anatomical site(s) each participant marked on the body chart as having pain. Once data collection was complete, the data between the two coders were compared, and if there was a lack of consensus a meeting was held with two additional team members to resolve the discrepancies.

### Data analysis

Data cleaning involved removing all demographics and clinical variables that had greater than 20% of data-points missing (ancestry, urban or rural study site, diabetes mellitus, active TB infection, current treatment for TB, shingles, previous exposure to stavudine, and current exposure to stavudine). In addition, no participants indicated that they had pain in the upper back (excluding the thoracic spine) and therefore this body site was dropped from all analyses. Two participants were removed from the analysis set because of improbably high CD4 T-cell counts (2000 and 7300 cells/mm^−3^), which we assumed were transcription errors at the time of the original data collection.

Summary data on the frequency of pain at each body site are reported as proportions, with the precision of point estimates given as 95% confidence intervals (CIs). Summary data on the total number of sites of pain per individual are reported as the median count, with the precision of the point estimates given as 95% CIs of the median, and the spread of the data reported as interquartile ranges (IQRs). All confidence intervals were calculated by the percentile method using bootstrapping (replicates = 999). Fisher’s Exact tests were used to detect for sex differences in pain at each body part. The Holm method was used to correct for multiple comparisons.

Mixed-effects negative binomial regression for over-dispersed count data was used to assess the effect of age (years), sex (male/female), CD4 T-cell count (cells/mm^3^), currently on ART (yes/no), level of education (primary, secondary, tertiary), and employment status (unemployed, part-time employment, full-time employment, other) on the number of pain sites. Study site was included as a random effect. However, because the fit was singular, we reanalysed the data as a fixed effect model only. The full model was compared to a null model using the likelihood ratio test.

All analyses were performed using R version 3.6.3^32^ with the following packages: boot,^33,34^ knitr,^35,36,37^ lme4,^38^ MASS,^39^ patchwork,^40^ skimr,^41^ and packages from the tidyverse.^42^ All data, analysis scripts, and supplementary files (Supplements 1, 2, and 3) are available at: https://github.com/kamermanpr/pain-sites.git. A Docker image with the environment required to run the scripts is available at: https://hub.docker.com/r/kamermanpr/pain-sites.

### Ethical considerations

Ethical approval was obtained from the Human Research Ethics Committee (Medical) of the University of the Witwatersrand for the following studies: study site 1 and 5 (both covered under ethics clearance number M140877), study site 2 (Clearance number: M041112), study site 3 (Clearance number: M140538). Ethical approval for study site 4 was obtained from the Faculty of Health Sciences Research Ethics Committee of the University of Cape Town (REC reference: 420 2007) and additionally, the Province of the Western Cape Department of Health (reference: 19/18/RP12/2009) as is required for studies in the Western Cape of South Africa.

## Results

### Demographics

Table 1 (and Supplement 1 [https://github.com/kamermanpr/pain-sites.git]) shows demographic and HIV disease-related statistics for the total group and for each study site. Across the study sites, the median age across the study sites ranged from a minimum of 31 years to a maximum of 45 years (study sites 4 and 5, respectively), with the total group having a median age of 36 (IQR: 31–42) years. Of all 596 participants 81% (481) were female, but this statistic was skewed by a single study (study site 4) that was restricted to women participants only. For studies where males and females were recruited, the proportion of female participants ranged from 65% (39/60) to 88% (44/50) (study sites 1 and 5, respectively). Overall, the majority of participants were unemployed (60%, 330/547), with the proportion of unemployed participants ranging from 44% (22/50) at study site 5 to 67% (99/148) at study site 4. Seventy per cent (70%, 395/559) of the total group had attained a secondary level of schooling (Grades 8 to 12). Tertiary education ranged from 4% (8/212, study site 2) to 27% (13/48, study site 5). Seventy-eight percent (78%, 460/591) of all participants were on ART, with the study site 2 having the lowest proportion on ART (55%, 130/236), whilst 100% (49/49) of participants at study site 5 were on ART. The median CD4 T-cell count across the study sites ranged from 186 to 435 cells/mm^−3^ (study sites 2 and 5, respectively), with the count for the entire group being 261 (IQR: 155–432) cell/mm^−3^. Although the data are not shown in Table 1 because many of the data were missing, the prevalence of diabetes in the total group was 4% (16/358) and TB was 30% (110/371). The mean (standard deviation [s.d.]) score for the BDI indicating depressive symptoms was 18 (10), which falls into the category of mild depression,^43^ (*n* = 251/596).

**TABLE 1:** Demographic and human immunodeficiency virus disease-related data for the total group and for each study site†.

Variable	Total (*n* = 596)					Site 1 (*n* = 60)					Site 2 (*n* = 239)					Site 3 (*n* = 99)					Site 4 (*n* = 148)					Site 5 (*n* = 50)				
	*n*	%	*n* missing	Median	IQR	*n*	%	*n* missing	Median	IQR	*n*	%	*n* missing	Median	IQR	*n*	%	*n* missing	Median	IQR	*n*	%	*n* missing	Median	IQR	*n*	%	*n* missing	Median	IQR
**Age (years)**	-	-	8	36	31–42	-	-	0	39	34–42	-	-	6	36	31–41	-	-	0	42	35–49	-	-	2	31	27–34	-	-	0	45	37–53
**Female**	481	81	0	-	-	39	65	0	-	-	184	77	0	-	-	66	67	0	-	-	148	100	0	-	-	44	88	0	-	-
**Employment**																														
Unemployed	330	60	-	-	-	36	60	-	-	-	126	66	-	-	-	47	47	-	-	-	99	67	-	-	-	22	44	-	-	-
Part-time	52	10	-	-	-	6	10	-	-	-	9	5	-	-	-	16	16	-	-	-	14	9	-	-	-	7	14	-	-	-
Full-time	131	24	-	-	-	17	28	-	-	-	55	29	-	-	-	35	35	-	-	-	4	3	-	-	-	20	40	-	-	-
Other‡	34	6	49	-	-	1	2	0	-	-	0	0	49	-	-	1	1	0	-	-	31	21	0	-	-	1	2	0	-	-
**Education**																														
Primary (grade: 1–7)	99	18	-	-	-	9	16	-	-	-	44	21	-	-	-	19	20	-	-	-	18	12	-	-	-	9	19	-	-	-
Secondary (grade: 8–12)	395	71	-	-	-	40	69	-	-	-	160	75	-	-	-	61	64	-	-	-	108	74	-	-	-	26	54	-	-	-
Tertiary§	65	12	37	-	-	9	16	2	-	-	8	4	27	-	-	15	16	4	-	-	20	14	2	-	-	13	27	2	-	-
**Currently on ART**	460	78	5	-	-	58	97	0	-	-	130	55	3	-	-	95	97	1	-	-	128	86	0	-	-	49	100	1	-	-
**CD4 T-cells** (cells/mm^−3^)	-	-	99	261	155–432	-	-	18	375	256–577	-	-	37	185	104–309	-	-	16	405	257–651	-	-	4	270	174–415	-	-	24	435	291–582

Note: Citation site 1 - Unpublished; citation site 2 - Mphahlele et al., 2012; citation sites 3 and 5 - Wadley et al., 2016; citation site 4 - Parker et al., 2016. Location sites 1, 2 3 and 5 - Johannesburg, Gauteng; location site 4 - Cape Town, Western Cape.

ART, antiretroviral therapy; IQR, interquartile range.

† , Percentages may not sum to 100% because of rounding;

‡ , Other: students and individuals receiving state grants (child, old-age, disability);

§ , Tertiary: any post-school qualification.

### Location of body sites with pain

**FIGURE 1:**
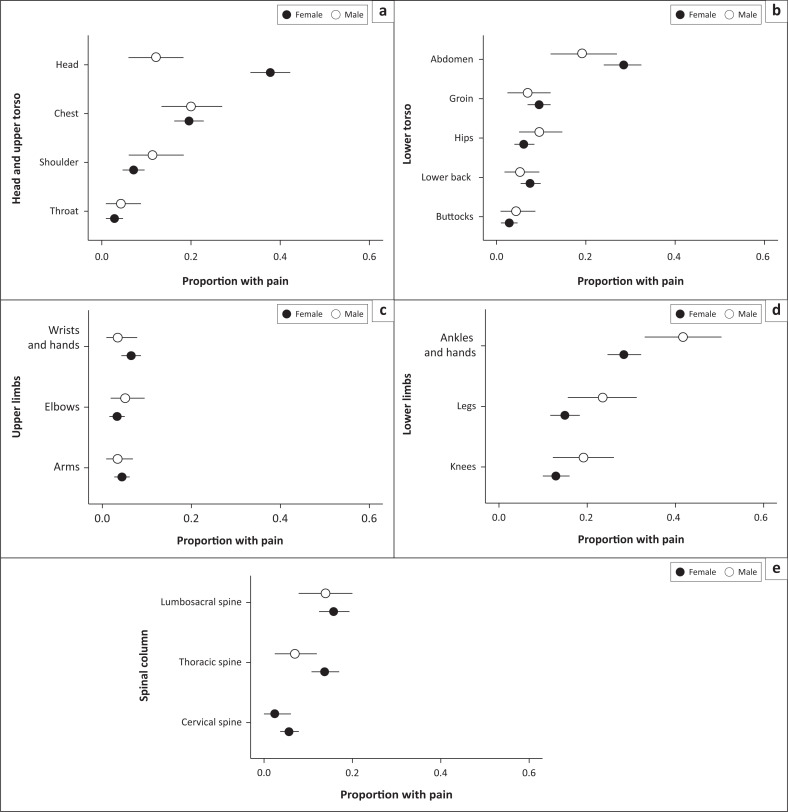
The proportion (95% confidence interval) of male and female participants with pain at 18 body sites grouped into five body regions: (a) Head and upper torso, (b) lower torso, (c) upper limbs, (d) lower limbs and (e) spinal column.

Figure 1 shows the proportion of males and females with pain at each of the 18 body sites (the same data are tabulated in Supplement 2, section 5.2 [https://github.com/kamermanpr/pain-sites.git]). The five most common body sites affected by pain in both males and females were the head (point estimate: 33%, 195/596), ankles and feet (31%, 184/596), abdomen (27%, 159/596), chest (20%, 117/596), and legs (excluding the knee) (17%, 99/596). Out of the 18 body sites examined, 10 had pain frequencies below 10% (throat, shoulder, arms, elbows, wrists & hands, lower back/flanks, cervical spine, groin, hips, and buttocks), with the five (5) least affected body sites being the upper back (excluding the thoracic spine; 0%, 0/596), throat (3%, 19/596), buttocks (3%, 19/596), arms (excluding the elbows; 4%, 25/596), and elbows (4%, 22/596). After correcting for multiple comparisons, males were less likely to experience headache than females (Fisher’s exact text, odds ratio [OR] = 0.23, 95% CI: 0.12 – 0.42, *p* = 0.000) but other body sites were experienced similarly between the sexes (Supplement 2, Section 5.4 [https://github.com/kamermanpr/pain-sites.git]).

### Number of pain sites

**FIGURE 2:**
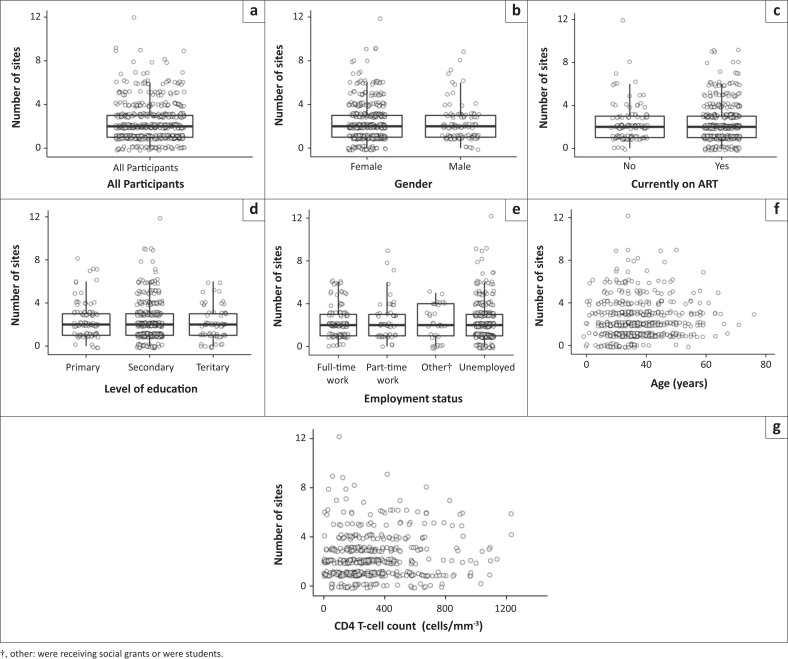
Number of pain sites stratified by sex, antiretroviral therapy (ART) exposure, level of education, employment status, age, and most recent CD4 T-cell count. For sex, ART exposure, level of education, and employment status the data are presented as Tukey box-and-whisker plots with individual data-points overlayed. Age and CD4 T-cell count data are presented as scatterplots of individual participant data. In all cases, and for the purpose of plotting only, individual-level data-points have been ‘jittered’ by adding a small amount of random noise: (a) All participants (*N* = 599), (b) sex (*n* = 599), (c) currently on ART (*n* = 594), (d) level of education (*n* = 562), (e) employment status (*n* = 550), (f) age (*I* = 591) and; (g) recent CD4 T-cell count (*n* = 500).

The median number of pain sites per participant was 2 (IQR: 1 to 3). One participant had 12 body sites affected by pain. Figure 2 shows the distribution of the number of pain sites according to sex, currently on ART, age, most recent CD4 T-cell count, level of education, and employment status; the fixed effects in the negative binomial regression modelling. The plots show no systematic variation in the number of pain counts by each variable, and these findings are backed-up by our modelling, in which the full model was not significantly different from the null model (Likelihood Ratio [LR] statistic = 13.12, *df* = 9, *p* = 0.157). Coefficients for the fixed effects of the model are reported in section 2.4.2 of Supplement 3 (https://github.com/kamermanpr/pain-sites.git). Tuberculosis, diabetes and BDI score were not included in the model as data missingness exceeded 20% (Supplement 1 [https://github.com/kamermanpr/pain-sites.git]). Univariate analysis, however, suggested no relationship between diabetes status and number of pain sites (Wilcoxon rank sum test, *p* = 0.105) or TB infection and pain sites (Wilcoxon rank sum test, *p* = 0.237). There was a weak positive relationship between BDI score and number of pain sites (Spearman’s rho = 0.15, *p* = 0.018) (Supplement 4 [https://github.com/kamermanpr/pain-sites.git]).

## Discussion

In this retrospective analysis of South Africans living with HIV and pain, we investigated whether there were sex differences in the location and number of pain sites. Secondly, we investigated if there were other demographic and disease-related differences in the number of pain sites. Contrary to our hypothesis, headache was the only site of pain that differed between the male and female participants and was less frequent in men than in women (12% in men vs. 38% in women). Whilst migraine headaches are twice as common in women from general populations, other forms of headache are as common in both sexes.^44^ It is possible that there was a high proportion of migraine headache that led to the sex difference in our cohort. We cannot confirm this, however, as we did not have detail of the headaches recorded. A high proportion of migraine may be unlikely as the Global Burden of Disease study reported that migraine only accounted for approximately one-fifth of headaches in Africa.^45^ Indeed, in a Ugandan study of 333 PLWH, migraine headache was seen in 19% of the cohort.^46^ Despite this relatively low prevalence of migraine in Ugandan PLWH, female sex and depression associated with having headache on multivariate analysis.^46^ It is possible that factors related to HIV contribute to the sex difference in general headaches seen here and in the Ugandan study.

Whilst pain sites were otherwise similar between South African men and women LWH, these sites differed from several recent studies from other countries. For example, headache was found in only 6% (38/638) of American PLWH accessing primary care^17^ and 18% (7/38) of individuals with acute pain in another cohort of American PLWH^47^ versus 33% in this study and 28% in a Ugandan study of PLWH.^46^ Indeed, the most common locations of pain here contrast to the United States (US) studies of PLWH,^11,17^ which reported spinal pain, joint pains and muscle aches as most common. In a United Kingdom (UK) study,^48^ pain sites were also dissimilar with the upper legs, back and shoulders being the most common pain sites. Our findings suggest that locations of pain sites cannot be generalised between cultures.

Pain in the ankle and feet was the second most common site of pain reported (31% of participants). Human immunodeficiency virus neuropathy is a common cause of ankle and foot pain in PLWH.^16,49^ Risk factors for neuropathy include previous or current TB, isoniazid treatment for TB without pyridoxine supplementation, alcohol abuse and nutritional deficiencies.^50,51,52,53^ Additionally, the studies included in this analysis were conducted in the era of stavudine-based ART, which is known to be neurotoxic^54^ and so may have contributed to the prevalence of ankle and foot pain here too. Our model indicated that increased foot and ankle pain was seen in those who were older, which fits with other studies of HIV neuropathy.^54^ First line antiretroviral regimens have changed since the time of data collection to include less neurotoxic drugs and the incidence of developing peripheral neuropathy within six-months of initiating ART has reduced from 41% to 17%.^50,55^ The continued existence of HIV neuropathy in non-stavudine-exposed cohorts seems to be independent of age^50^ and has been suggested to be a result of immune dysregulation.^50,56,57^

The median number of pain sites was 2. Indeed, 56% of men and 62% of women had more than one pain site. Number of pain sites has not been frequently reported in studies of pain in PLWH. In the few studies that have reported number of pain sites, the median has been 1–2^21,23,48,58^ and was greater in PLWH compared to individuals not living with HIV.^48^ The number of pain sites here was not associated with age, CD4 T-cell count, level of education or employment status. A recent study found that lower level of education, ART exposure, higher CD4 T-cell but longer duration of immunosuppression were independently associated with pain being widespread, as opposed to being regional.^48^ Associations have been found between multisite pain and the presence of inflammation.^11^ Given the results of this analysis and earlier studies, mechanism-based studies of pain in HIV should include exploration of inflammation.

### Limitations

There were several limitations to this analysis. The studies included here were not designed to assess sex differences in location and number of pain sites and thus this was a post-hoc analysis. As such, our findings may be coincidental and require replication in studies specifically designed to assess sex differences in location and number of pain sites. Whilst univariate analysis suggested no relationship between TB and diabetes, and number of pain sites, there was significant missing data (TB = 48%, diabetes = 40%). We did not include variables with > 20% missingness in our regression analysis and so the lack of association with TB and diabetes needs to be regarded with caution. An additional factor with significant missing data was the BDI (missing data = 58%) because not all studies had measured depression or had used different scales. Depression and anxiety frequently associate with pain, including in PLWH.^59,60^ Univariate analysis indicated a weak relationship between number of pain sites and BDI score but because of high data missingness, this relationship must also be regarded with caution. We assessed associations between demographic and disease-related factors and number of pain sites but not location of pain sites. We chose not to analyse location of pain sites because there would have been so many comparisons. To get around this issue, we recommend future studies look at factors associating with specific pain sites. For example, the association of TB and diabetes with foot and ankle pain in this new era of less neurotoxic ART. Additionally, we would recommend assessing associations between depression and headache, chest and abdominal pain; which have been described as sites of somatisation of mental health symptoms in other cohorts.^61,62^

Further limitations include that the pain-related picture we presented was limited. For example, we did not include measures of pain intensity or functional impairment because they have already been reported. Another limitation is that the Cape Town cohort (study site 4) included only women aged 18–40 years old. This may have lowered the average number of pain sites for women as they would have presented with fewer age-related complaints.^63^ If anything, we may have presented an under-representation of the problem rather than an over-representation. There was no control group of individuals from the same socioeconomic grouping and geographical areas living without HIV. There is preliminary evidence, however, that the prevalence of, and associations with pain are similar between South Africans both living with and without HIV.^60,64^

## Conclusion

Our objectives were to investigate whether there were sex differences in the location and number of pain sites and secondly, whether there were other demographic and disease-related differences in the number of pain sites. This is the first analysis to compare location and number of pain sites in young South African PLWH and pain. Other than a lower prevalence of headache in men, the locations of pain sites were similar between the sexes. The locations of pain sites were different from recent international studies suggesting that research into pain in PLWH cannot necessarily be generalised across cultures. We found that men experienced the same number of pain sites as women. The multiple pain sites support theories of inflammation, and/or immune dysregulation as mechanisms for pain in PLWH. To elucidate on the mechanisms contributing to pain in PLWH, we recommend that future studies explore the interrelationships between inflammation, comorbid conditions (such as depression, anxiety, TB and diabetes), and the number and location of pain sites in PLWH.

## References

[1] Kamerman PR, Bradshaw D, Laubscher R, et al. Almost 1 in 5 South African adults have chronic pain: A prevalence study conducted in a large nationally representative sample. Pain. 2020;161(7):1629–1635. 10.1097/j.pain.0000000000001844 32102020

[2] Racine M, Tousignant-Laflamme Y, Kloda LA, Dion D, Dupuis G, Choinière M. A systematic literature review of 10 years of research on sex/gender and experimental pain perception – Part 1: Are there really differences between women and men? Pain. 2012 Mar 1;153(3):602–618. 10.1016/j.pain.2011.11.025 22192712

[3] GBD 2017 DALYs and HALE Collaborators. Global, regional, and national disability-adjusted life-years (DALYs) for 359 diseases and injuries and healthy life expectancy (HALE) for 195 countries and territories, 1990–2017: A systematic analysis for the Global Burden of Disease Study 2017 – The Lancet. 2018;392:1859–1922. 10.1016/S0140-6736(18)32335-3 PMC6252083 30415748

[4] Fillingim RB, King CD, Ribeiro-Dasilva MC, Rahim-Williams B, Riley JL. Sex, gender, and pain: A review of recent clinical and experimental findings. J Pain. 2009 May;10(5):447–485. 10.1016/j.jpain.2008.12.001 19411059 PMC2677686

[5] Bartley EJ, Fillingim RB. Sex differences in pain: A brief review of clinical and experimental findings. Br J Anaesth. 2013 Jul;111(1):52–58. 10.1093/bja/aet127 23794645 PMC3690315

[6] Ruau D, Liu LY, Clark JD, Angst MS, Butte AJ. Sex differences in reported pain across 11,000 patients captured in electronic medical records. J Pain. 2012 Mar;13(3):228–234. 10.1016/j.jpain.2011.11.002 22245360 PMC3293998

[7] Gerdle B, Björk J, Cöster L, Henriksson K, Henriksson C, Bengtsson A. Prevalence of widespread pain and associations with work status: a population study. BMC Musculoskelet Disord. 2008 Jul 15;9:102. 10.1186/1471-2474-9-102 18627605 PMC2488345

[8] Català E, Reig E, Artés M, Aliaga L, López JS, Segú JL. Prevalence of pain in the Spanish population: telephone survey in 5000 homes. Eur J Pain Lond Engl. 2002;6(2):133–140. 10.1053/eujp.2001.0310 11900473

[9] Parker R, Stein DJ, Jelsma J. Pain in people living with HIV/AIDS: a systematic review. J Int AIDS Soc. 2014;17:18719. 10.7448/IAS.17.1.18719 24560338 PMC3929991

[10] UNAIDS. Global HIV statistics fact sheet [homepage on the Internet]. 2021 [cited 2021 Oct 4]. Available from: chrome-extension://efaidnbmnnnibpcajpcglclefindmkaj/viewer.html?pdfurl=https%3A%2F%2Fwww.unaids.org%2Fsites%2Fdefault%2Ffiles%2Fmedia_asset%2FUNAIDS_FactSheet_en.pdf&clen=179065&chunk=true

[11] Merlin JS, Westfall AO, Heath SL, et al. Brief report: IL-1β levels are associated with chronic multisite pain in people living with HIV. J Acquir Immune Defic Syndr. 2017;75(4):e99–e103. 10.1097/QAI.0000000000001377 28328552 PMC5484722

[12] Yuan S-B, Shi Y, Chen J, et al. Gp120 in the pathogenesis of human immunodeficiency virus-associated pain. Ann Neurol. 2014 Jun;75(6):837–850. 10.1002/ana.24139 24633867 PMC4077969

[13] Navis A, Jiao J, George MC, Simpson D, Robinson-Papp J. Comorbid pain syndromes in HIV-associated peripheral neuropathy. Pain Med. 2018 Jul 1;19(7):1445–1450. 29024980 10.1093/pm/pnx129

[14] Madden VJ, Parker R, Goodin BR. Chronic pain in people with HIV: A common comorbidity and threat to quality of life. Pain Manag. 2020 Jul;10(4):253–260. 10.2217/pmt-2020-0004 32484065 PMC7421257

[15] Merlin JS, Westfall AO, Chamot E, et al. Pain is independently associated with impaired physical function in HIV-infected patients. Pain Med. 2013 Dec;14(12):1985–1993. 10.1111/pme.12255 24119077 PMC3886835

[16] Ellis RJ, Rosario D, Clifford DB, et al. Continued high prevalence and adverse clinical impact of human immunodeficiency virus-associated sensory neuropathy in the era of combination antiretroviral therapy: the CHARTER Study. Arch Neurol. 2010 May;67(5):552–558. 10.1001/archneurol.2010.76 20457954 PMC3924778

[17] Jiao JM, So E, Jebakumar J, George MC, Simpson DM, Robinson-Papp J. Chronic pain disorders in HIV primary care: clinical characteristics and association with healthcare utilization. Pain. 2016 Apr;157(4):931–937. 10.1097/j.pain.0000000000000462 26683238

[18] Miaskowski C, Penko JM, Guzman D, Mattson JE, Bangsberg DR, Kushel MB. Occurrence and characteristics of chronic pain in a community-based cohort of indigent adults living with HIV infection. J Pain Off J Am Pain Soc. 2011 Sep;12(9):1004–1016. 10.1016/j.jpain.2011.04.002 PMC3164738 21684218

[19] Hitchcock SA, Meyer HP, Gwyther E. Neuropathic pain in AIDS patients prior to antiretroviral therapy. South Afr Med J. 2008 Nov;98(11):889–892. 19177897

[20] Aouizerat BE, Miaskowski CA, Gay C, et al. Risk factors and symptoms associated with pain in HIV-infected adults. J Assoc Nurses AIDS Care. 2010;21(2):125–133. 10.1016/j.jana.2009.10.003 20116299 PMC2832225

[21] Breitbart W, McDonald MV, Rosenfeld B, et al. Pain in ambulatory AIDS patients. I: Pain characteristics and medical correlates. Pain. 1996 Dec;68(2–3):315–321. 10.1016/S0304-3959(96)03215-0 9121820

[22] Hashmi JA, Davis KD. Deconstructing sex differences in pain sensitivity. Pain. 2014 Jan;155(1):10–13. 23891901 10.1016/j.pain.2013.07.039

[23] Mphahlele NR, Mitchell D, Kamerman PR. Pain in ambulatory HIV-positive South Africans. Eur J Pain. 2012 Mar;16(3):447–458. 10.1002/j.1532-2149.2011.00031.x 22337525

[24] Parker R, Jelsma J, Stein DJ. Managing pain in women living with HIV/AIDS: A randomized controlled trial testing the effect of a six-week peer-led exercise and education intervention. J Nerv Ment Dis. 2016;204(9):665–672. 10.1097/NMD.0000000000000506 27002748

[25] Parker R, Madden VJ, Devan D, et al. Barriers to implementing clinical trials on nonpharmacological treatments in developing countries: lessons learnt from addressing pain in HIV. Pain Rep. 2019;4(6):e783. 10.1097/PR9.0000000000000783 31984291 PMC6903358

[26] Wadley AL, Mitchell D, Kamerman PR. Resilience does not explain the dissociation between chronic pain and physical activity in South Africans living with HIV. PeerJ. 2016 Sep 13;4:e2464. 27672513 10.7717/peerj.2464 PMC5028784

[27] Wadley AL, Pincus T, Evangeli M. A preliminary analysis of the association between perceived stigma and HIV-related pain in South Africans living with HIV. Afr J Prim Health Care Fam Med. 2019;11(1):1–5. 10.4102/phcfm.v11i1.1647 PMC6407439 30843417

[28] Balady GJ, Chaitman B, Driscoll D, et al. Recommendations for cardiovascular screening, staffing, and emergency policies at health/fitness facilities. Circulation. 1998 Jun 9;97(22):2283–2293. 9631884 10.1161/01.cir.97.22.2283

[29] Cleeland CS, Ryan KM. Pain assessment: Global use of the Brief Pain Inventory. Ann Acad Med Singap. 1994 Mar;23(2):129–138. 8080219

[30] Daut RL, Cleeland CS, Flanery RC. Development of the Wisconsin Brief Pain Questionnaire to assess pain in cancer and other diseases. Pain. 1983 Oct;17(2):197–210. 10.1016/0304-3959(83)90143-4 6646795

[31] Beck AT, Steer RA, Brown GK. Manual for the Beck Depression Inventory – Second Edition. San Antonia, TX: The Psychological Corporation; 1996.

[32] R Core Team. R: A language and environment for statistical computing. (Version 3.6.3) [Computer software]. R Foundation for Statistical Computing. Vienna: R Foundation for Statistical Computing. [cited 2021 Oct 16]. Available from: https://www.R-project.org/

[33] Canty A, Ripley B. boot: Bootstrap R (S-Plus) Functions (Version 1.3-24) [Computer software]. [cited 2021 Oct 16]. Available from: https://cran.r-project.org/web/packages/boot/

[34] Davison A, Hinkley D. Bootstrap methods and their applications [homepage on the Internet]. Cambridge: Cambridge University Press; 1997 [cited 2021 Oct 16]. Available from: http://statwww.epfl.ch/davison/BMA/

[35] Xie Y. knitr: A Comprehensive tool for reproducible research in R. In: Stodden V, Leisch F, Peng R, editors. Implementing reproducible computational research [homepage on the Internet]. Chapman and Hall/CRC; 2014 [cited 2021 Oct 16]. Available from: http://www.crcpress.com/product/isbn/9781466561595

[36] Xie Y. Dynamic documents with R and knitr [homepage on the Internet]. Chapman/CRC Press; 2015 [cited 2021 Oct 16], p. 294. Available from: https://yihui.name/knitr/

[37] Xie Y. knitr: A general-purpose package for dynamic report generation in R (Version 1.28) [Computer software]. 2020 [cited 2021 Oct 16]. Available from: https://yihui.name/knitr/

[38] Bates D, Mächler M, Bolker B, Walker S. Fitting linear mixed-effects models using lme4. J Stat Softw. 2015;67:1–48. 10.18637/jss.v067.i01

[39] Venables W, Ripley B. Modern applied statistics with S (Fourth) [homepage on the Internet]. Springer. [cited 2021 Oct 16]. Available from: http://www.stats.ox.ac.uk/pub/MASS4

[40] Pedersen T. patchwork: The composer of ggplots (Version 1.0.0) [Computer software]. 2019 [cited 2021 Oct 16]. Available from: https://CRAN.R-project.org/package=patchwork

[41] Quinn M, McNamara A, Arino de la Rubia E, Zhu H, Ellis S. skimr: compact and flexible summaries of data. (Version 2.1) [Computer software]. 2019 [cited 2021 Oct 16] Available from: https://CRAN.R-project.org/package=skimr

[42] Wickham H, Averick M, Bryan J, et al. Welcome to the Tidyverse. J Open Source Softw. 2019 Nov 21;4(43):1686. 10.21105/joss.01686

[43] Dozois DJA, Dobson KS, Ahnberg JL. A psychometric evaluation of the Beck Depression Inventory–II. 1998;10(2):83–89. 10.1037/1040-3590.10.2.83

[44] Buse DC, Loder EW, Gorman JA, et al. Sex differences in the prevalence, symptoms, and associated features of migraine, probable migraine and other severe headache: results of the American Migraine Prevalence and Prevention (AMPP) Study. Headache. 2013 Sep;53(8):1278–1299. 10.1111/head.12150 23808666

[45] Saylor D, Steiner TJ. The global burden of headache. Semin Neurol. 38(2):182–190. 10.1055/s-0038-1646946 29791944

[46] Sohail S, Nakigozi G, Anok A, et al. Headache prevalence and its functional impact among HIV-infected adults in rural Rakai District, Uganda. J Neurovirol. 2019 Apr;25(2):248–253. 30607892 10.1007/s13365-018-0710-9 PMC6506364

[47] Uebelacker LA, Weisberg RB, Herman DS, Bailey GL, Pinkston-Camp MM, Stein MD. Chronic pain in HIV-infected patients: Relationship to depression, substance use, and mental health and pain treatment. Pain Med. 2015 Oct;16(10):1870–1881. 10.1111/pme.12799 26119642 PMC4486324

[48] Sabin CA, Harding R, Bagkeris E, et al. The predictors of pain extent in people living with HIV. AIDS. 2020 Nov 15;34(14):2071–2079. 10.1097/QAD.0000000000002660 32773481

[49] Ellis RJ, Diaz M, Sacktor N, et al. Predictors of worsening neuropathy and neuropathic pain after 12 years in people with HIV. Ann Clin Transl Neurol. 2020 Jul;7(7):1166–1173. 10.1002/acn3.51097 32619341 PMC7359117

[50] Pillay P, Wadley AL, Cherry CL, Karstaedt AS, Kamerman PR. Clinical diagnosis of sensory neuropathy in HIV patients treated with tenofovir: A 6-month follow-up study. J Peripher Nerv Syst. 2019 Dec;24(4):304–313. 10.1111/jns.12349 31587421

[51] Van der Watt JJ, Benatar MG, Harrison TB, Carrara H, Heckmann JM. Isoniazid exposure and pyridoxine levels in human immunodeficiency virus associated distal sensory neuropathy. Int J Tuberc Lung Dis. 2015 Nov;19(11):1312–1319. 10.5588/ijtld.15.0467 26467583

[52] Gwathmey KG, Grogan J. Nutritional neuropathies. Muscle Nerve. 2020 Jul;62(1):13–29. 10.1002/mus.26783 31837157

[53] Julian T, Glascow N, Syeed R, Zis P. Alcohol-related peripheral neuropathy: A systematic review and meta-analysis. J Neurol. 2019 Dec;266(12):2907–2919. 30467601 10.1007/s00415-018-9123-1 PMC6851213

[54] Kamerman PR, Moss PJ, Weber J, Wallace VCJ, Rice ASC, Huang W. Pathogenesis of HIV-associated sensory neuropathy: evidence from in vivo and in vitro experimental models. J Peripher Nerv Syst. 2012 Mar;17(1):19–31. 10.1111/j.1529-8027.2012.00373.x 22462664

[55] Pillay P. The incidence of peripheral neuropathy in HIV-positive individuals on highly active antiretroviral therapy (HAART) [homepage on the Internet] [thesis]. 2014 [cited 2021 Feb 10]. Available from: http://wiredspace.wits.ac.za/handle/10539/13726

[56] Gui W-S, Wei X, Mai C-L, et al. Interleukin-1β overproduction is a common cause for neuropathic pain, memory deficit, and depression following peripheral nerve injury in rodents. Mol Pain. 2016;12:1744806916646784. 10.1177/1744806916646784 27175012 PMC4956151

[57] Shi Y, Gelman BB, Lisinicchia JG, Tang S-J. Chronic-pain-associated astrocytic reaction in the spinal cord dorsal horn of human immunodeficiency virus-infected patients. J Neurosci. 2012 Aug 8;32(32):10833–10840. 10.1523/JNEUROSCI.5628-11.2012 22875918 PMC3470811

[58] Breitbart W, Rosenfeld B, Passik S, Kaim M, Funesti-Esch J, Stein K. A comparison of pain report and adequacy of analgesic therapy in ambulatory AIDS patients with and without a history of substance abuse. Pain. 1997 Aug;72(1–2):235–243. 9272808 10.1016/s0304-3959(97)00039-0

[59] Scott W, Arkuter C, Kioskli K, et al. Psychosocial factors associated with persistent pain in people with HIV: a systematic review with meta-analysis. Pain. 2018 Dec;159(12):2461–2476. 10.1097/j.pain.0000000000001369 30130299 PMC6250281

[60] Wadley AL, Lazarus E, Gray GE, Mitchell D, Kamerman PR. Pain in clients attending a South African voluntary counseling and testing center was frequent and extensive but did not depend on HIV status. J Acquir Immune Defic Syndr. 2020 Feb 1;83(2):181–188. 10.1097/QAI.0000000000002248 31929406

[61] Andersen L, Kagee A, O’Cleirigh C, Safren S, Joska J. Understanding the experience and manifestation of depression in people living with HIV/AIDS in South Africa. AIDS Care. 2015 Jan 2;27(1):59–62. 10.1080/09540121.2014.951306 PMC4241601 25303372

[62] De Heer EW, Gerrits MMJG, Beekman ATF, et al. The association of depression and anxiety with pain: A study from NESDA. PLoS One. 2014 Oct 15;9(10):e106907. 10.1371/journal.pone.0106907 25330004 PMC4198088

[63] Rustøen T, Wahl AK, Hanestad BR, Lerdal A, Paul S, Miaskowski C. Age and the experience of chronic pain: differences in health and quality of life among younger, middle-aged, and older adults. Clin J Pain. 2005 Dec;21(6):513–523. 10.1097/01.ajp.0000146217.31780.ef 16215337

[64] Wadley AL, Iacovides S, Roche J, Scheuermaier K, Venter WDF, Vos AG. Working nights and activity associate with chronic pain in a large cohort of Southern African truckers. PLoS One. 2020;15(12):e0243366. 10.1371/journal.pone.0243366 33270793 PMC7714191

